# Functional, molecular, and digital measurements of biological age

**DOI:** 10.1172/JCI205777

**Published:** 2026-06-15

**Authors:** Baljash S. Cheema, Bedirhan Boztepe, Moses O. Awofolaju, Mallory S. Hubbard, William B. Marcus, Frank J. Palella, Mohamed Abdel-Mohsen, David M. Liebovitz, Manjot K. Gill, R. James Cotton, John T. Wilkins, Douglas E. Vaughan

**Affiliations:** 1 Northwestern University Feinberg School of Medicine, Chicago, Illinois, USA.; 2 Shirley Ryan AbilityLab, Chicago, Illinois, USA.

## Abstract

The reality of an aging population demands a deeper understanding of aging as a biological process, rather than as a chronological descriptor. Chronological age poorly captures interindividual heterogeneity in physiological and functional decline, disease susceptibility, and mortality risk. In contrast, biological age encompasses deterioration at the molecular, cellular, tissue, organ, functional, and organismal levels and provides insight into why two individuals with the same chronological age exhibit differences in physiological function, disease susceptibility, and mortality risk. While early models of biological age relied on functional markers or composite scores derived largely from longitudinal cohort studies, more recent models integrate molecular profiling with machine learning to ascertain biological aging trajectories. In parallel, new artificial intelligence tools have been applied to various imaging modalities and other forms of complex data to elucidate latent patterns and estimate biological age. In this state-of-the-art Review, we explore historical and modern approaches to estimating biological age and highlight key conceptual, technical, and translational challenges that remain unresolved. As geroscience-guided interventions are incorporated into clinical evaluations, robust and accurate interpretable measures of biological aging are crucial to ascertain treatment effects in clinical trials.

## Introduction

Modern medicine, together with major improvements in public health, sanitation, nutrition, and environmental and social conditions, has driven extraordinary progress in promoting health, treating disease, and extending lifespan. Alongside declining fertility and childbearing rates, these advances have contributed to a global demographic shift, with an unprecedented proportion of the population expected to be aged 60 years or over in the coming decades (1). This aging trend has profound implications for healthcare systems, as advancing chronological age (CA) is the primary risk factor for nearly all chronic diseases, including cardiovascular disease (CVD), neurocognitive disease, and cancer (2, 3). Therefore, our infrastructure for managing chronic diseases, and our ability to deliver sustainable, equitable, timely, and appropriate healthcare, may become increasingly strained (4, 5). The growing burden of aging-related disease necessitates a deeper understanding of aging itself, not only as a demographic trend but also as a biological process that can be quantified, characterized, and potentially modified.

**Figure 1:**
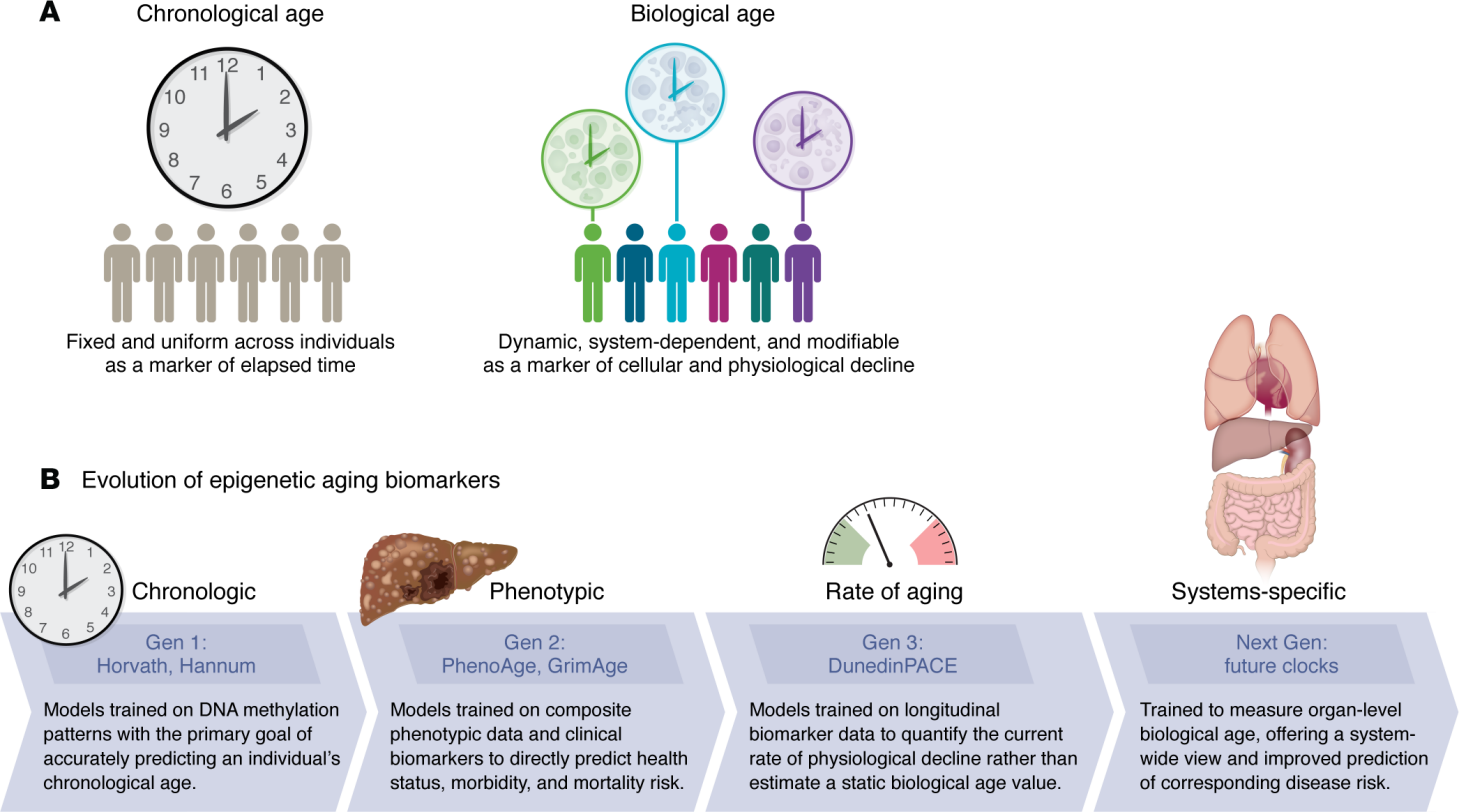
The distinction between CA and BA and the progression of epigenetic clocks through multiple generations. (**A**) Chronological age reflects a uniform measure of time, whereas biological age reflects cellular and physiological decline. (**B**) First-generation clocks (e.g., Horvath, Hannum) were trained to predict chronological age. Second-generation clocks (e.g., PhenoAge, GrimAge) focus on phenotypic health and mortality risk. Third-generation clocks (e.g., DunedinPACE) measure the current rate of biological aging. The next generation of clocks aims to measure organ-specific biological age.

While CA, defined as the number of years since birth, is routinely used to predict incident disease and risk stratify patients, it fails to explain the substantial variability observed in physical function, disease susceptibility, resilience, and mortality among individuals of the same age and similar established risk factors, such as sex, smoking, adiposity, and cardiometabolic health (6–10). These observations have driven the emergence of the concept of biological age (BA), a more nuanced measure that encompasses the cumulative effects of aging on an individual, influenced by environmental exposures, health behaviors, genetic predisposition, and chance events (Figure 1A). Some measures of BA outperform CA in predicting future health states (11–13). Early efforts to measure BA used individual factors or composite indices derived from longitudinal cohort studies investigating health, well-being, and survival (14–19). With the identification of hallmarks of aging and improvements in molecular profiling, the field has pivoted toward high-dimensional estimates of BA using data derived from genetics, epigenetics, transcriptomic profiling, proteomics, and other approaches (20, 21). Most recently, machine learning (ML) and artificial intelligence (AI) have enabled the discovery of complex, latent patterns across large datasets, revealing meaningful signatures of BA (22–26).

Clearly defining and adjudicating measures of BA are crucial for evaluating interventions, whether based on health behavior changes, supplements, or pharmaceuticals, that aim to slow aging and extend health span (13, 27–30). The concept of health span, defined as years lived free of disease and disability, predates contemporary BA frameworks and has long informed public health efforts, with CVD serving as a key exemplar (31, 32). This pursuit is grounded in the geroscience hypothesis, which posits that the molecular and cellular hallmarks of aging are the causal link between CA and age-associated dysfunction, frailty, and the development of chronic disease (33); therefore, interventions that modulate these underlying mechanisms may mitigate the consequences of aging (9, 34, 35). While animal models have led to the identification of promising interventional candidate measures to promote longevity and health span, rigorous validation through clinical trials in humans remains a major unmet need (36).

In this state-of-the-art narrative Review, we synthesize the major approaches to quantifying BA, spanning clinical metrics, molecular profiling platforms, and emerging AI-driven models (Table 1). We evaluate their conceptual foundations, methodological strengths, practical limitations, interpretability, and translational relevance. We further recognize that many of these tools remain primarily research measures rather than established instruments for routine clinical care, and we highlight key gaps that must be addressed before broader clinical adoption. Together, these perspectives illuminate both the progress made and the challenges that remain as the field moves toward more standardized, interpretable, and biologically grounded measures of aging.

## Traditional approaches: physical performance, organ function, and blood-based biomarkers

Early BA estimates relied on simple clinical measures drawn from longitudinal aging cohorts: investigators compared individuals with age- and sex-matched reference groups and interpreted deviations as accelerated or delayed aging, sometimes converting these differences into “biological years.” Some groups also combined these clinical variables into composite indices to summarize physiological aging (6, 15, 37–40), often overlapping with constructs of frailty and physiological reserve (41). These tools are easy to collect, clinically intuitive, and capture meaningful variation in physiological reserve and vulnerability to stressors, but they provide limited insight into the cellular and molecular drivers of aging.

### Physical performance biomarkers.

Physical function was one of the earliest and most pragmatic approaches to estimating BA, in part because declines in mobility and strength are widely recognized features of aging and closely track individuals’ lived experience of functional loss. Gait speed is among the best-studied metrics. In a pooled analysis across nine cohort studies (1986–2000) including more than 34,000 adults aged 65 and over, each 0.1 m/s higher gait speed was associated with a 10%–12% lower mortality risk (42). Its relevance extends into midlife: in the Dunedin birth cohort, slower gait at age 45 tracked with weaker physical performance, older facial appearance, and structural brain changes (e.g., smaller brain volume, greater cortical thinning), suggesting that gait captures early, system-level aging rather than late functional decline alone (43). Because of its reproducibility across cohorts and substantial prognostic value, some have referred to gait speed as a “sixth vital sign,” although its routine clinical adoption has been limited (44).

Grip strength, measured by dynamometer squeeze, is another robust marker of systemic aging (45). In the large, multinational PURE study of 139,691 adults, each 5 kg decrease in grip strength was associated with a 16% higher risk of all-cause mortality (46). A meta-analysis of more than three million participants across 42 cohorts showed similar patterns, linking weaker grip to higher mortality as well as CVD, cancer, and stroke risk (47). Other performance measures, including the timed-up-and-go test, the short physical performance battery, and the sit-to-stand test, also show that reduced strength, balance, and mobility are tightly coupled to adverse health outcomes (48–50). For example, unipedal stance time, a measure of balance and stability, declines more rapidly with aging than gait speed or grip strength (51). Together, these findings illustrate that mobility measures do not simply quantify functional status but may act as integrative readouts of longevity. Newer AI-based tools now utilized in research settings, including video-derived gait analysis and computer vision–based methods, may improve the precision and scalability of these established functional measures (52, 53), offering a promising future direction toward defining a more granular “movement age.”

Sensory function, including vision, hearing, and olfaction, offers a scalable organ-level readout that may reflect systemic aging beyond isolated end-organ loss. In large population-based cohorts, poorer multisensory function is associated with worse physical and cognitive performance and higher short-term mortality, even after adjustment for major confounders (54). Across longitudinal analyses, mortality risk increases with both the number and severity of sensory impairments, suggesting a cumulative multisensory aging signal (55, 56).

### Organ function biomarkers.

Endothelial function declines with age, with impaired vasodilation reflecting reduced nitric oxide bioavailability and accumulated oxidative stress (57). Flow-mediated dilation, an ultrasound-based measure of conduit artery function, is strongly linked to cardiovascular (CV) events (58) and provides a noninvasive window into vascular aging (59). Because endothelial health integrates metabolic, inflammatory, and hemodynamic inputs, it can be seen as a physiologic interface of systemic aging rather than reflecting vessel-specific pathology alone.

Arterial stiffness reflects age-related alterations in elastin-collagen balance, cross-linking, inflammation, and smooth muscle stiffening. It contributes to isolated systolic hypertension and elevated pulse pressure (PP) (60) and meets criteria for a prognostic CV biomarker (61). Aortic pulse-wave velocity is the most established measure: across longitudinal cohorts, higher pulse-wave velocity is associated with roughly twofold increases in CV and all-cause mortality after adjustment (62). This suggests that vascular stiffening is not merely a CV change but a proxy for global loss of tissue elasticity and repair capacity. Central PP, which better reflects central organ load than brachial BP, also correlates more strongly with subclinical organ damage; each 10 mmHg increase in central PP is associated with an approximately 14% higher CVD risk (63).

Cardiac physiological measures capture cumulative effects of aging on autonomic regulation, myocardial relaxation, and CV reserve (64). Heart rate variability (HRV), derived from beat-to-beat (RR interval) fluctuations, captures autonomic control of cardiac rhythm. Mendelian randomization analyses indicate that lower genetically predicted HRV causally increases CV risk, positioning HRV as a marker of declining autonomic resilience with age (65). The echocardiographic ratio (E/e′), an estimate of left ventricular filling pressure, independently predicts mortality; CV events including myocardial infarction, stroke, and heart failure; or hospitalizations for any cause, with a HR of approximately 1.05–1.06 per 1-unit increase in E/e′ (66). Reduced early-diastolic velocity (e′), indicative of impaired left ventricular relaxation, likewise predicts adverse outcomes and captures age-related diastolic dysfunction even in the absence of overt disease (67). Maximal oxygen uptake, providing an integrated measure of CV, pulmonary, and muscular performance, is one of the strongest indicators of whole-body physiological reserve. In the HUNT3 Fitness Study cohort (healthy adults), maximal oxygen uptake declined progressively approximately 6%–8% per decade, with both submaximal and peak exercise models capturing this trajectory across adulthood (68). Higher aerobic capacity is strongly associated with higher long-term survival; in the 46-year Copenhagen cohort, midlife cardiorespiratory fitness was closely tied to improved longevity (69).

Pulmonary function declines with CA as lung tissue loses elasticity, respiratory muscles weaken, and small airways narrow, reducing ventilatory capacity and gas-exchange efficiency (70). Peak expiratory flow and forced vital capacity show consistent age-related reductions, and lower forced vital capacity is associated with higher cardiometabolic risk even in individuals without overt lung disease (39). Forced expiratory volume in 1-second declines by approximately 20–50 mL per year in longitudinal studies (71, 72) and correlates with CV and mortality risk, indicating that respiratory aging may mirror systemic biological aging rather than isolated organ decline (39).

### Blood-based biomarkers.

Natriuretic peptides (BNP and NT-proBNP) are established markers of cardiac strain and long-term CV risk, even at levels far below heart failure diagnostic thresholds (73). Data from several aging-focused cohorts support the notion that NT-proBNP rises steadily with age and serves as a BA indicator. In a cohort of 1,079 older adults, NT-proBNP was the single most predictive age-related biomarker, outperforming routine labs for predicting 7-year mortality, enabling derivation of a “proBNPage” that tracked BA more closely than CA (74). Later work showed that higher proBNPage maps onto previously discussed broader aging phenotypes (e.g., slower gait, weaker grip, cognitive complaints) often more strongly than CA (75), and the Rugao Longevity and Ageing Study similarly linked higher BNP levels to these phenotypes in CVD-free older adults (76). A similar pattern emerges with cystatin C, which although viewed as a renal marker, behaves more like a systemic health parameter across aging cohorts. Even in healthy individuals without kidney impairment, cystatin C rises nonlinearly with age, indicating that it tracks intrinsic renal aging and broader systemic decline (77). Meta-analytic evidence shows that elevated cystatin C predicts long-term all-cause (HR ~1.7–1.9) and CV mortality (HR ~2.0), consistently outperforming creatinine-based indices (78). Higher levels are also linked to fewer disease-free years and a greater risk of transitioning to physical or cognitive disability (79).

Plasminogen activator inhibitor-1 (PAI-1) reflects a related but distinct aspect of aging biology. PAI-1 is a serine protease inhibitor central to fibrinolysis that increases in both chronological and stress-induced aging and is a core component of the senescence-associated secretory phenotype (80). In a Berne Amish kindred, carriers of a rare *SERPINE1* (the gene encoding PAI-1) loss-of-function allele had longer telomeres, lower fasting insulin, less type 2 diabetes mellitus, and a longer lifespan (81). Larger cohort data align with this pattern: midlife women in the SWAN study with higher PAI-1 showed adverse metabolic and CV profiles (82).

These senescence-linked changes converge with a second central axis of biological aging: chronic, low-grade inflammation (inflammaging) (83). Inflammaging is marked by elevated circulating cytokines, chemokines, and acute-phase reactants related to both immune system dysfunction and accumulated molecular damage over time and is consistently associated with higher and earlier onset morbidity and mortality in older adults (84, 85). IL-6, in particular, correlates with endothelial dysfunction and is linked to atherogenic processes independent of serum lipid levels (86). Complementing IL-6, high-sensitivity C-reactive protein reflects downstream inflammatory signaling and, in contemporary statin-treated populations, has been a stronger predictor of future CV events and mortality than LDL-C, underscoring the concept of residual inflammatory risk (87). Within the TNF axis, circulating soluble TNF receptors (sTNFR1 and sTNFR2) are often used as stable biomarkers of chronic TNF pathway activation and have been associated with CV events and mortality in prospective studies (88–90). Multimarker approaches suggest that coordinated elevation across IL-6, high-sensitivity C-reactive protein, and TNF pathway readouts identifies a higher-risk inflammatory phenotype than any single analyte alone (91, 92). Metabolic markers capture complementary dimensions of aging biology: growth differentiation factor 15, a key senescence-associated secretory phenotype component, rises with metabolic dysregulation and declining renal function, mirroring multisystem physiologic burden (93). By contrast, the IGF-1 axis has been linked to vascular and endothelial function (94). HbA1c also increases with age even in metabolically healthy individuals and predicts mortality and functional decline across cohorts, highlighting its role as a broad marker of metabolic aging rather than merely insulin resistance in type 2 diabetes mellitus or glycemic control (95–98). One explanation for this observation is that HbA1c partly reflects erythrocyte turnover and glycation kinetics, both of which shift with aging independent of glucose exposure (97, 99, 100).

Although individual biomarkers provide valuable information about specific physiological systems, they cannot capture the multisystem decline that characterizes biological aging. One of the earliest composite BA indices was the Klemera–Doubal method, which shows a strong correlation with CA as well as a 7%–9% higher risk of mortality during follow-up, independent of traditional risk factors (6, 16). Approaches such as PhenoAge incorporated clinical biomarkers selected for their associations with mortality, improving predictive accuracy; each 1-year increase in PhenoAge was associated with a 9% higher mortality risk (HR 1.09) in the derivation cohort. For individuals in the highest quintile, mortality rates were approximately 3.3-fold higher than in the lowest quintile despite similar CA (101). Complementing these cross-sectional composites, longitudinal measures such as the Dunedin Pace of Aging metric quantified the slope of decline across 18 biomarkers spanning metabolic, CV, renal, hepatic, immune, and pulmonary systems, measured repeatedly over 20 years in the Dunedin cohort. Individuals aging “one biological year per chronological year” showed a mean Pace of Aging of 1.0, whereas persons aging faster exhibited values ≥ 1.2, consistent with approximately20% faster organ system deterioration and substantially poorer physical and cognitive function by midlife (102).

Closely related to composite measures of biological aging, resilience and intrinsic capacity have emerged as central functional endpoints in geroscience. Resilience reflects an individual’s ability to withstand and recover from physiological and psychosocial stressors, while intrinsic capacity, introduced by WHO, captures the integrated reserve of physical and mental function across five key domains: cognition, locomotion, vitality, psychological well-being, and sensory function (103, 104). Both constructs prioritize functional status over disease burden, reflecting a broader shift from disease-centered toward function-centered assessment focused on maintaining independence, health span, and recovery capacity. As multidomain and dynamic constructs that focus on adaptive reserve rather than accumulated pathology, resilience and intrinsic capacity are increasingly recognized as relevant outcome measures for aging research and interventional trials (105).

Finally, recent work has furthered the notion of composite aging metrics of inflammaging. One example is the inflammatory aging clock (iAge), derived using deep learning (DL) on circulating inflammatory profiles, which tracks multimorbidity, immunosenescence, frailty, and CV aging and highlights CXCL9 as a major contributor linked to vascular dysfunction and arterial stiffness (106).

## Molecular approaches: epigenetics, proteomics, and emerging diagnostics

Advances in molecular biology have enabled aging to be studied not merely as a clinical phenotype but as a quantifiable biological process driven by specific cellular mechanisms (21, 107, 108). Following the original description of the nine cellular and molecular hallmarks of aging, later expanded in subsequent revisions and summarized in Table 2, researchers have increasingly sought to measure these processes with reliability and precision (83, 109, 110). Advances in high-throughput molecular profiling and computational modeling have made it possible to interrogate thousands of molecular features simultaneously, revealing reproducible signatures of biological aging across tissues and populations. Among these, DNA methylation–based “epigenetic clocks” have emerged as some of the most robust predictors of morbidity and mortality, linking changes in CpG methylation to age-related decline (111–116). Parallel efforts in proteomics, metabolomics, glycomics, and inflammatory profiling have expanded this landscape, generating integrative frameworks that capture the multidimensional biology of aging.

### Epigenetic clocks.

Some of the earliest epigenetic clocks, including those proposed by Horvath (multitissue composite age estimation) and Hannum (single-tissue age estimation), were based mainly on supervised ML prediction models incorporating specific CpG methylation sites (21, 117–119). These first-generation epigenetic clocks employed a linear model that predicted CA from DNA methylation patterns. The Horvath and Hannum clocks were highly correlated with CA (Pearson’s *r* = 0.96 and 0.72, respectively), and most importantly age gap, the difference in estimated age and one’s CA, predicted all-cause mortality despite not including more relevant risk factors in the prediction model such as BMI, smoking, alcohol consumption, activity level, education, and other comorbidities (120, 121). These first-generation clocks were not without disadvantages. Technical variation (sample preparation and processing) and biological variation (interindividual variability inherent to certain CpG methylation sites) often led to inconsistency with repeat measurements (122, 123). One study reported that technical replication of the Horvath clock yielded high deviation, with a median of 3.6 years (124). Both of these clocks demonstrated weak associations with clinical biomarkers (e.g., BP, glucose metabolism) and diminished correspondence with tissue-specific ages, limitations prompting the development of newer clocks better equipped to predict morbidity and mortality (125).

Second-generation epigenetic clocks, such as DNAm PhenoAge and GrimAge, used linear models based around DNA methylation patterns to predict biological aging phenotypes rather than just CA (101, 125, 126). DNAm PhenoAge was trained using a weighted composite of 10 clinical characteristics to first estimate PhenoAge, as mentioned previously. This phenotype-driven weighted composite was then used to construct a prediction model based on CpG methylation sites and showed meaningful differences in disease and mortality among individuals of the same CA (101, 125). GrimAge was trained on CpG markers acting as surrogates for smoking history and plasma protein levels to predict time to death (125, 126). One study comparing all four clocks in a single cohort (*n =* 490) with a mean age of 62.2 years (SD = 8.3), found that PhenoAge (*r* = 0.85, SD = 9.6) and GrimAge (*r* = 0.81, SD = 7.6) had stronger age correlations than the Horvath and Hannum clocks (*r* = 0.74, SD = 11) (125). Another study showed that while first-generation clocks had no association with specific measures of functional health and cognitive performance, PhenoAge and GrimAge were associated with outcomes like weaker grip strength, worse lung function, and slower mental speed (107). With this more phenotype-driven approach, both second-generation clocks have outperformed previous clocks in predicting age-related health outcomes and lifespan, with GrimAge being the most predictive epigenetic clock for all-cause mortality (125, 127).

The novel DunedinPACE clock, termed a third-generation clock, utilizes the Pace of Aging metric mentioned earlier alongside DNA methylation patterns to derive a single, point-in-time blood test accounting for the biological patterns differentiating individuals with faster versus slower rates of aging (15, 128). Individuals with a higher DunedinPACE score exhibit more rapid aging-related decline in both physical and cognitive functions. While its predictions are consistent with other clocks, its focus on the rate of aging gives it distinct advantages in avoiding the survival bias inherent in clocks trained on older cohorts, and it can better distinguish ongoing aging-related decline from preexisting deficits developed in childhood (128). Although it gives less weight to mortality than second-generation clocks like GrimAge, it still provides improved incremental predictive value for morbidity and mortality (128, 129). This specific focus makes DunedinPACE a particularly valuable tool for investigating the effects of behavioral or pharmaceutical interventions on the aging process itself.

Epigenetic aging biomarkers (Figure 1B) continue to be refined, including the recently developed organ systems–based CpG methylation clock, Systems Age, which quantifies ages in 11 distinct organ systems using data from a single blood draw while providing a composite age score reflecting overall multisystem aging (130). Biomarkers are also being developed to leverage the multifactorial nature of aging by moving beyond CpG methylation. Another clock, OMICmAge, implements a regression model integrating multiple molecular profiling frameworks — proteomics, glycomics, and metabolomics — to capture aging hallmarks more directly influenced by biological and environmental phenomena. Preliminary results from its preprint publication suggest OMICmAge is precise, reliable over time, and accounts for changes in immune cell subsets that can influence age estimation (131).

### Proteomic clocks.

As the end products of epigenetic and transcriptional regulation, proteins directly influence phenotype by integrating system-wide biological processes, making them major contributors to the physiological and pathological outcomes of aging (132, 133). Advancements in assay technologies now enable the measurement of thousands of circulating proteins from small blood samples in a highly reproducible manner, increasing the potential of biomarker discovery with a proteomics focus (132, 134). Proteomic clocks leverage these capabilities, with some regression models trained to predict CA or mortality as a surrogate for BA, though they are usually limited by training with smaller sets of proteins known to be associated with specific biological aging processes (106, 132, 133, 135). Recent work aimed to develop a proteomic aging clock (PAC), based on various blood-borne proteins (*n =* 2,920), to predict all-cause mortality as an estimate of BA similar to second-generation epigenetic clocks (136, 137). The PAC provided age-adjusted predictions of all-cause mortality and the onset of various diseases with a relatively robust correlation with CA (*r* = 0.77), demonstrating stronger associations than other biomarkers such as DNAm PhenoAge, BioAge, and short leukocyte telomere length (136). However, PAC was not established to have predictive power for disease- or organ-specific BA estimation, though this has been explored in other work with a proteomics focus that may provide greater insight (137, 138).

### Additional molecular clocks.

A large population study leveraged IgG Fc N-glycosylation profiles to derive a glycan-based estimator of age, commonly referred to as GlycanAge (139). A minimal three-variable index dominated by glycan structures, including FA2B, FA2G2, and FA2BG2, explained approximately 58% of the variance in CA, with a correlation between chronological and predicted age of about *r* = 0.76 and a residual SD of approximately 9.7 years. Performance was lower but directionally consistent in independent cohorts (139). Because IgG Fc glycosylation is a functional regulator of antibody effector activity and inflammatory potential, age-associated shifts in these glycans may provide mechanistic links between immune remodeling and systemic inflammaging. Importantly, the residual variance in GlycanAge after accounting for CA correlated with physiological and biochemical traits, supporting its interpretation as a proxy for aspects of biological aging, although the model itself was trained primarily on CA (139).

Additional work has been done investigating how quantifying end products of metabolism could inform an association with CA and health outcomes. One such clock, MileAge, was developed by training multiple ML algorithms on plasma metabolites (*n =* 168) to predict mortality and health span (140). Performance differed based on the algorithm used (nonlinear vs. linear models), yielding moderate correlations with CA (*r* = 0.36–0.59 and a mean absolute error of 5.31–6.36 years). Though MileAge proved to be an adequate predictor of CA and mortality, other metabolomic clocks have demonstrated improved predictive power, further highlighting the potential of metabolomic aging measurement (140–142).

## Digital approaches: AI for latent pattern detection

**Figure 2:**
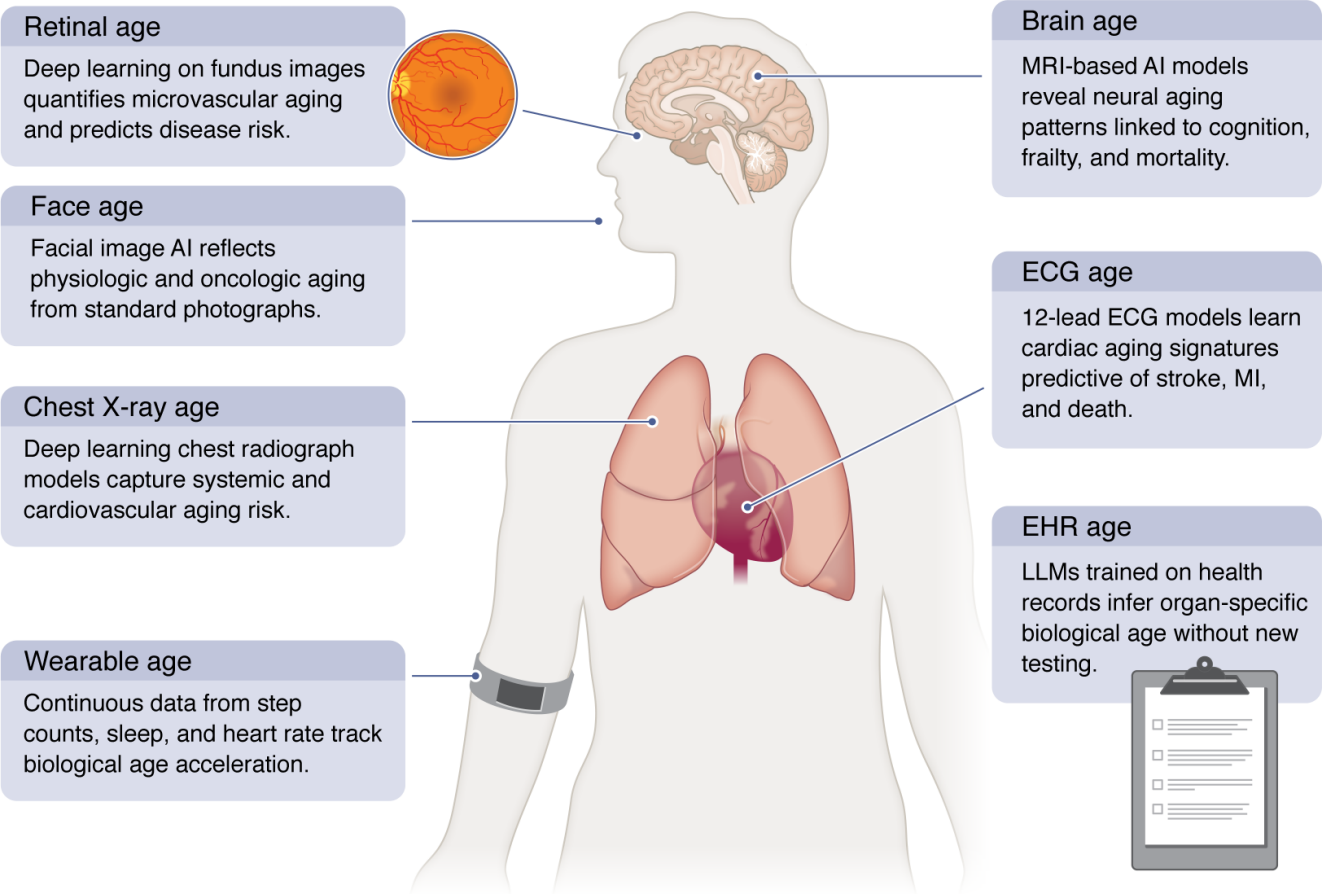
AI-derived BA from multimodal digital data. Models estimate age from imaging, electrophysiology, wearable signals, and clinical text to capture organ-level physiological change. MI, myocardial infarction.

The exponential growth of digital health data combined with increases in computational power and advanced AI algorithms specifically utilizing DL has led to new opportunities to quantify BA (143). Whereas traditional and molecular biomarkers of disease rely on predefined variables, AI systems can learn latent representations directly from complex, multimodal data, ranging from imaging and electrocardiography to genomics and clinical records (144, 145). In many cases, these models are trained to predict CA, and the residual age gap is interpreted as a proxy for accelerated or decelerated biological aging. These methods extend beyond human-interpretable features to capture nonlinear and multidimensional signatures and may be particularly well-suited to studying the cumulative physiological decline across all organ systems with age. Foundation or large AI models trained on broad datasets are increasingly available publicly, enabling task adaptation and helping to democratize access to AI while supporting robust performance across external cohorts (146, 147). These AI systems span multiple data modalities, including imaging, electrophysiology, wearable sensors, and clinical text, each offering a distinct window into BA (Figure 2).

### ECG-age.

An ECG captures dynamic voltage changes across the chest and remains one of the most informative, widely used tools for assessing cardiac structure, electrophysiology, and overall CV health. Although clinical interpretation relies on review of a compressed, standardized visual representation of the raw waveform, DL systems can analyze the full, high-dimensional signal and detect subtle, temporally distributed features that are inaccessible to human readers and can reveal previously unrecognized associations with cardiac disease (148). In aging research, deep neural networks trained to predict CA from ECG data have been used to estimate “ECG-age” (149). The gap between the ECG-age and CA has been shown to correlate with hard CV outcomes, including stroke, myocardial infarction, and death, in several independent studies (150, 151). In the largest ECG-age study to date, a deep residual network was trained on 1,558,415 patients from the Brazilian CODE cohort to predict age from 12-lead ECGs. Patients whose ECG-age exceeded CA by >8 years had a higher risk of all-cause mortality during a median follow-up of 3.4 years (HR 1.79, 95% CI 1.69–1.90), whereas those whose ECG-age was >8 years younger had lower mortality (HR 0.78, 95% CI 0.74–0.83), even after adjustment for age and sex. Similar HRs were replicated in two external cohorts (ELSA-Brasil, *n =* 14,236; SaMi-Trop, *n =* 1,631), supporting the interpretation of ECG-age as a digital composite measure of CV-BA rather than a simple age regressor (150). These findings suggest that AI-derived ECG-age reflects the cumulative physiological wear of the CV system and may serve as a readily accessible digital biomarker of biological aging, especially with new foundation models being developed (147).

### Retinal-age.

In a similar approach to deriving ECG-age, high-resolution color fundus photographic images of the retinal microvasculature have been analyzed with DL models to estimate a “retinal-age” (152). The difference in retinal-age and CA has been linked to important health outcomes including mortality, CVD, chronic kidney disease, and dementia (153–160). For example, a DL model trained on retinal fundus images from UK Biobank participants estimated a 2% higher hazard of all-cause mortality per 1-year increase in retinal age gap over follow-up (HR 1.02, 95% CI 1.00–1.03), after multivariable adjustment, again illustrating that a single image-derived age gap can function as a composite health and aging score (153). While these studies have been limited by potential selection bias from image-quality filtering and have shown worse model performance in external validation cohorts (154, 161), there is the potential to improve on this work by leveraging other retinal imaging techniques, such as optical coherence tomography (OCT), OCT angiography, and ultra-wide-field imaging, and to use foundation models as a starting point for future work (146, 161, 162).

### Brain-age, facial aging, and other imaging-based models.

Generalizing further, a growing body of research demonstrates that diverse imaging modalities harbor detectable signatures of biological aging. Brain MRI, for example, has been used to derive “brain-age,” which allows for age gap measures that strongly correlate with cognitive decline, frailty, and mortality risk across populations (163, 164). In a cohort of 669 older adults from the Lothian Birth Cohort 1936, a ML model trained on brain volumes produced a mean absolute error of 4.2 years for age prediction; individuals with an older-than-expected brain (positive brain-predicted age difference [brain-PAD]) had higher mortality, with each additional year of brain-PAD associated with an approximately 6% increase in all-cause mortality risk (HR 1.06, 95% CI 1.01–1.10) after adjustment for CA and traditional risk factors (165). Chest radiography (CXR) offers a more accessible clinical alternative, enabling estimation of CXR-age that predicts all-cause and CV mortality independent of established risk factors (166, 167). Facial imaging represents yet another distinct pathway, one that does not rely on medical testing and may allow for opportunistic capture from photographs. FaceAge, a convolutional neural network (DL architecture optimized for image and signal analysis), was trained on 58,851 healthy individuals and validated in 4,906 patients with cancer, quantifying a facial age gap that is strongly prognostic. Across pan-cancer, thoracic radiotherapy, and palliative cohorts, each 10-year increase in FaceAge was associated with worse overall survival (e.g., pan-cancer, HR 1.151, *P* = 0.013, *n =* 4,906), and cancer patients on average appeared 4.79 years older than matched noncancer controls (*P* < 0.0001). Incorporating FaceAge improved clinicians’ short-term survival predictions in palliative radiotherapy (AUC 0.80 vs. 0.74; *P* < 0.0001), highlighting that even a facial photograph–derived age gap can operate as a composite frailty and BA signal (168).

### Large language models of clinical text.

Modern large language models (LLMs) trained on clinical text and health records are now demonstrating the ability to estimate BA-related measures without the need for imaging or high-dimensional molecular profiling (169). For example, Li et al. (169) developed a framework in which an LLM was prompted with standard health examination reports to predict both overall and organ-specific ages. They found that the LLM-predicted overall age achieved a concordance index of about 0.757 (95% CI 0.752–0.761) for all-cause mortality, outperforming traditional aging proxies such as telomere length, frailty index, epigenetic age scores, and models using ML. The LLM-derived age gap predicted all-cause death, and organ-specific age gaps also predicted corresponding disease categories better than classic ML models (169). This work demonstrates that LLMs are scalable for population-level phenotypic aging estimation because they rely on routinely collected clinical text from existing electronic health data.

Importantly, LLM-based age estimates should not be interpreted as mechanistic models of aging. Instead, they function as high-dimensional summary measures of clinical phenotype, integrating information across diagnoses, laboratory values, and documented health trajectories. It is not surprising that such models show strong associations and discrimination in mortality and disease outcomes, often exceeding those observed for single-modality aging metrics. Unlike proteomic or molecular clocks, however, LLM-derived age gaps do not directly identify upstream biological drivers of aging or specific pathways amenable to intervention; these models are best viewed as downstream tools for identifying individuals with “accelerated aging” phenotypes, complementary to mechanistic aging measures rather than substitutes for them.

## Gaps in knowledge

Despite substantial progress in the development of BA measures across clinical, molecular, and digital domains, several important gaps limit broader adoption and utility. First, the field lacks consensus on a unifying framework for what constitutes an adequate BA metric. At present, no broadly accepted standard exists, and creating one is beyond the scope of this review (28). Additionally, reliability, longitudinal stability, responsiveness to interventions, and feasibility across diverse healthcare settings are variably measured and rarely compared head-to-head. Furthermore, most BA metrics are supported primarily by cross-sectional and prognostic data, but there remains limited evidence that within-person changes over time reliably reflect aging rate, respond to interventions, or qualify as clinically meaningful surrogate endpoints. As a result, there is no consensus on the magnitude or clinical relevance of changes in BA measures in response to health behaviors or clinical interventions aimed at improving health (27). Standardized benchmarking datasets, shared evaluation criteria, and harmonized reporting standards will be essential to move from isolated clocks toward a more coherent approach to aging assessment. Additionally, it is important to distinguish biomarkers that predict outcomes, reflect accumulated disease burden or physiologic decline, and directly illuminate mechanisms of aging biology, as current evidence is strongest for the first two and more limited for the third.

Second, not all BA metrics are anchored in biology, with molecular clocks as examples of well-cited tools for BA assessment that often aggregate thousands of features into composite scores with limited interpretability (20). Digital and AI-derived clocks rely on high-dimensional representations that are even less transparent, complicating efforts to understand which pathways are being captured (170). Systematic efforts to link clock components to specific hallmarks of aging, tissue, or organ-specific processes and causal pathways are needed to move from association toward mechanism-informed measurement.

Third, a related unresolved challenge is distinguishing aging-related biological change from disease-induced organ dysfunction when interpreting BA estimates. Because aging and disease mechanisms are intertwined, elevated BA may reflect accelerated aging, subclinical pathology, established disease, reduced resilience, or a combination thereof, underscoring the need for contextual interpretation across these overlapping phenomena (3, 9, 27, 28).

Fourth, there is a paucity of comparative studies that directly evaluate traditional, molecular, and digital clocks within the same individuals (171). As a result, the degree of redundancy versus complementarity among different BA measures is poorly understood. It is hypothesized that epigenetic, proteomic, imaging-based, and digital clocks each capture distinct dimensions of aging, reflecting different organs, systems, or timescales; however, further research is needed to verify this and outline comprehensive approaches to BA estimation that are not redundant. This will likely require multimodal integration studies that combine these signals and formally assess their unique and shared contributions to risk prediction, functional decline, and resilience.

Finally, there is limited evidence on how BA metrics should be deployed in clinical settings, including (a) their role in clinical assessment and (b) how evidence of accelerated biological aging should inform clinical management decisions. Few studies have rigorously evaluated how BA responds to interventions known to modify aging-related pathways, how much change is biologically meaningful, or how BA measures should be incorporated into trial design. While the geroscience hypothesis posits that changes in BA measures should impact that aging process directly and may slow the progression of chronic disease development, this is a hypothesis that requires rigorous evaluation and testing in prospective clinical trials. These trials must give attention to cost, workflow integration, and health equity to demonstrate real-world value of BA estimates. Closing these gaps will require coordinated efforts across disciplines, shared infrastructure, and deliberate linkage between measurement science and intervention studies aimed at extending health span. Additional pitfalls in BA interpretation and suggested approaches to mitigate fallacies are provided in Table 3.

## Future research directions

**Figure 3:**
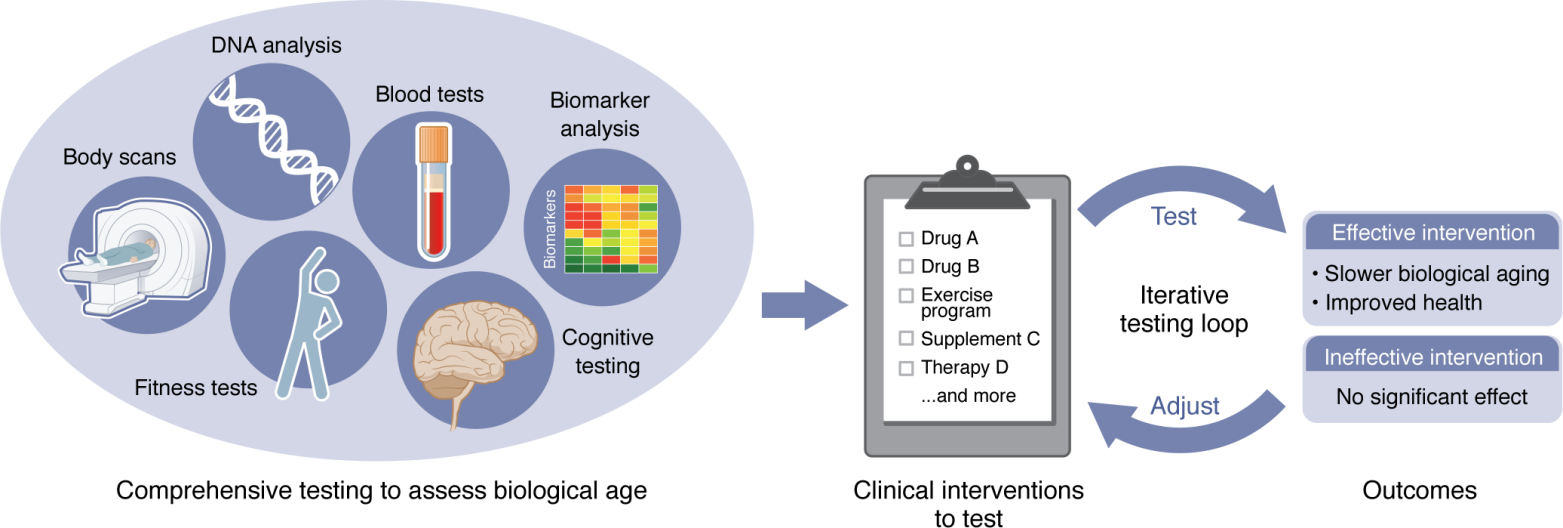
Conceptual framework for BA-guided intervention studies. Repeated BA assessments are used prospectively alongside targeted interventions to evaluate aging trajectories and intervention responsiveness.

Best practices for translating BA metrics into clinical practice and identifying interventions that improve the aging process will require detailed, objective, prospective human studies that use a comprehensive approach to BA estimation as a modifiable clinical endpoint alongside metrics of physical function and organ system–specific health that decline with aging. If an intervention truly impacts the aging process, consistent effects across multiple aging biomarkers, rather than isolated changes, are expected. As an initial step, harmonized, small-to-moderate scale signal-finding trials using a unified testing platform across multiple sites should test diverse approaches for enhancing health span and well-being across genetically and culturally diverse populations. This conceptual framework illustrates how repeated BA assessments, paired with targeted interventions, can be used longitudinally to track aging trajectories and treatment responsiveness, framing BA as a dynamic, modifiable construct rather than a static measurement (Figure 3). This approach to aging research can identify which interventions are most likely to result in reproducible, clinically meaningful changes that target aging biology and will inform design of subsequent phase III clinical trials. Throughout this process, attention to financial cost, workflow integration, regulatory acceptance, and equity must be considered to ensure that potential interventions are not only scientifically valid but feasible for clinical integration and generalizable to real-world healthcare systems.

BA metrics offer a promising path to quantify individual variation in aging, anticipate health outcomes beyond CA, and accelerate the evaluation of interventions that target underlying aging processes. At present, the field is characterized by methodological heterogeneity, limited comparative and longitudinal data, and sparse evidence from prospective intervention studies. Moving forward, progress will depend on converging toward consensus definitions, shared evaluation standards, and coordinated, multisite clinical observational and interventional trials that embed harmonized BA panels as endpoints. By aligning measurement science with mechanistic insight, rigorous trial design, and attention to implementation, BA can evolve from a research construct into a practical tool to guide prevention, personalize care, and ultimately extend health span.

## Author contributions

BSC, BB, MOA, MSH, WBM, FJP, MAM, DML, MKG, RJC, JTW, and DEV contributed meaningfully to conceptualizing the paper, creating an outline, writing the manuscript, drafting/editing tables and figures, and editing the final work. Order of co–first authors reflects their involvement in the project.

## Conflict of interest

BSC is an advisor with equity in Zoe Biosciences. FJP has received consulting fees and speaking honoraria from Gilead Sciences, ViiV Pharmaceuticals, Merck, and EMD Serono. MKG has served as a consultant for Genentech-Roche, Regeneron, and Kriya Therapeutics. RJC holds US patents 11,803,241 and 12,277,272 and US patent applications 16/588,202 and 14/534,163. DEV has served as a consultant for The Medicines Company and is a cofounder of Zoe Biosciences.

## Funding support

This work is the result of NIH funding, in whole or in part, and is subject to the NIH Public Access Policy. Through acceptance of this federal funding, the NIH has been given a right to make the work publicly available in PubMed Central.

- NIH grant R01HD114776 (to RJC).
- Research Accelerator Program of the Shirley Ryan AbilityLab (to RJC).
- National Heart, Lung, and Blood Institute grant R35HL171553 (to DEV).
- NIH grants R01AG092241, R01AI189353, R01AI165079, and R01AA031197 (to MAM).

## Figures and Tables

**Table 1:**
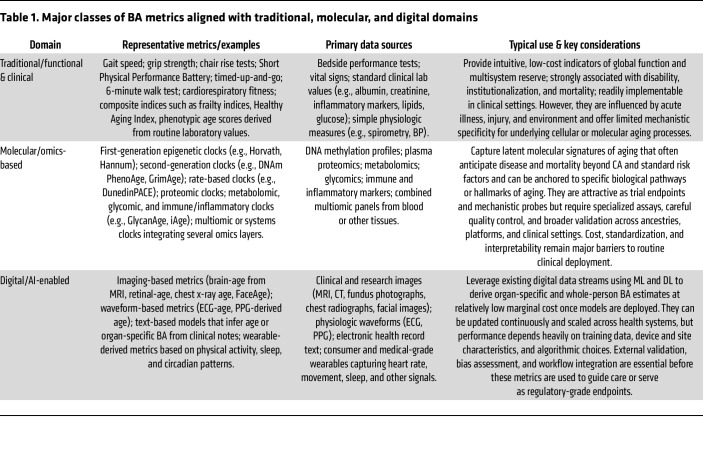
Major classes of BA metrics aligned with traditional, molecular, and digital domains

**Table 2:**
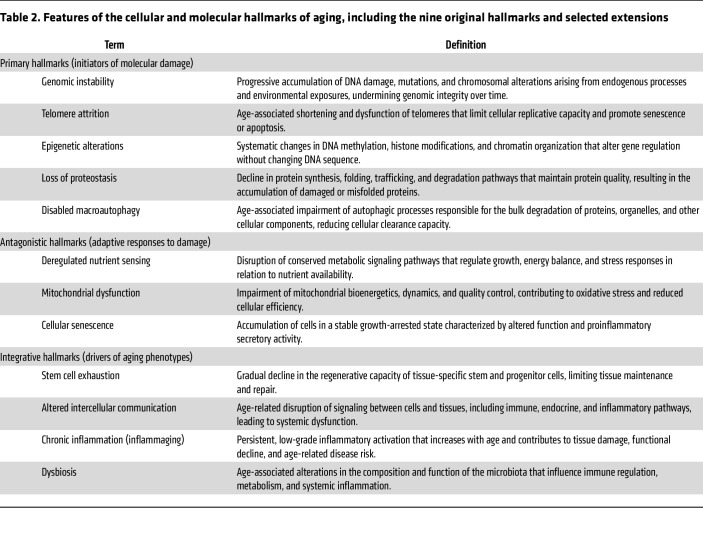
Features of the cellular and molecular hallmarks of aging, including the nine original hallmarks and selected extensions

**Table 3:**
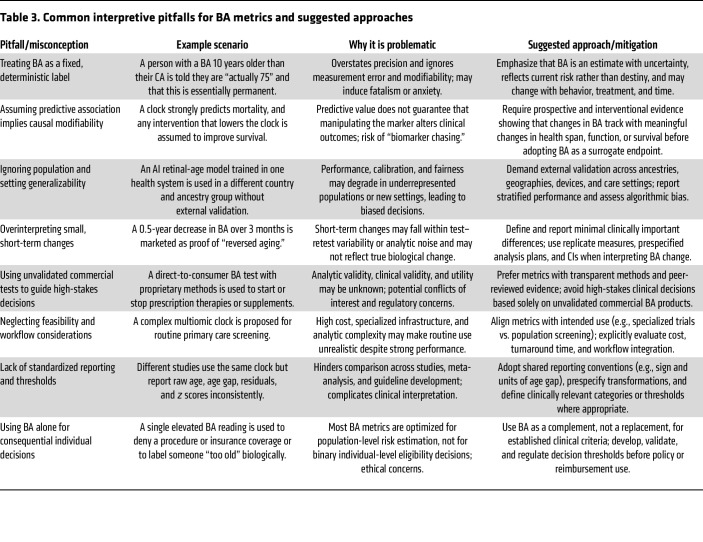
Common interpretive pitfalls for BA metrics and suggested approaches

## Version 1 06/15/2026

Electronic publication
